# Phase contrast mri measurement of e/a and e/e'

**DOI:** 10.1186/1532-429X-13-S1-P237

**Published:** 2011-02-02

**Authors:** Neil Chatterjee, Jeremy Collins, James Carr, Peter J Weale

**Affiliations:** 1 Northwestern University Feinberg School of Medicine, Chicago, IL, USA; 2 Northwestern University, Department of Radiology, Chicago, IL, USA; 3 Siemens Medical Solutions, Chicago, IL, USA

## Introduction

MRI is the accepted gold standard for assessment of left ventricular systolic function; however, no standards are available to assess diastolic function at MRI. E/A and E/e’ ratios are currently used in echocardiography to evaluate left ventricular diastolic function. Measuring these ratios with phase contrast MRI may provide a complementary approach to assessing left ventricular function.

## Purpose

To validate E/A and E/e’ ratios acquired with phase contrast MRI relative to established values using echocardiography.

## Methods

17 self-reported healthy volunteers were recruited under an IRB approved protocol. Ultra-fast phase contrast data was acquired on a 1.5T Siemens Aera using both breath-hold (30 frames per cardiac cycle) and free breathing (50 frames per cardiac cycle) paradigms. To measure e’ velocities, phase contrast data (Venc 25cm/s) was acquired in the short axis orientation at a slice position where the myocardium on the apical side of the valve ring was within the slice throughout the cardiac cycle. To measure E and A velocities, phase contrast data (Venc 80 cm/s) was acquired in a single slice parallel to the mitral valve annulus, positioned such that the slice stayed below the valve throughout the entire cardiac cycle. E and A velocities as well as septal and lateral e’ velocities were calculated using standard flow post-processing. 2 subjects were excluded from both analyses due to improper gating, and 4 additional subjects were excluded from the breath hold analysis due to too much noise to identify e’ velocities.

## Results

With free breathing, E/A was measured at 1.7 ± 0.5 (range 0.8 - 2.6), septum E/e’ was measured at 5.4 ± 1.5 (range 2.5 - 7.4), and lateral E/e’ was measured at 5.1 ± 1.7 (range 2.6 - 8.4). With breath hold, E/A was measured at 1.5 ± 0.6 (range 0.7 - 2.7), septum E/e’ was measured at 6.1 ± 2.2 (range 3.6 - 10.2), and lateral E/e’ was measured at 5.4 ± 2.1 (range 3.5 - 10.9). Subjects with E/e’ above 8.0 had normal left atrial size.

## Conclusions

Measured E/A and E/e’ values are within normal limits using cutoff values that have been published with echocardiography [1], suggesting that phase contrast MRI may provide a complementary approach to assessing left ventricular diastolic function.
